# Ecological Niche Changes of Two Sympatric Species (*Pennahia argentata* and *Larimichthys polyactis*) Revealed by Bulk and Compound Specific Isotope Analyses

**DOI:** 10.1002/ece3.72792

**Published:** 2025-12-26

**Authors:** Tae‐Sik Yu, Bohyung Choi, Yeonjung Lee, Ihn‐Sil Kwak

**Affiliations:** ^1^ Fisheries Science Institute Chonnam National University Yeosu‐si Republic of Korea; ^2^ Inland Fisheries Research Institute National Institute of Fisheries Science Geumsan‐gun Republic of Korea; ^3^ Ocean Climate Reponse & Ecosystem Research Department Korea Institute of Ocean Science and Technology Busan‐si Republic of Korea; ^4^ Department of Ocean Integrated Science Chonnam National University Yeosu‐si Republic of Korea

**Keywords:** compound‐specific stable isotope analysis, isotopic niche, stable isotope analysis, sympatric species, trophic position

## Abstract

Sympatric species in marine ecosystems often share habitats and food resources, leading to niche competition. We performed ecological niche width estimation based on bulk carbon (δ^13^C) and nitrogen (δ^15^N) isotopes and trophic position estimation via compound‐specific isotope analysis (CSIA) of amino acids to compare ecological interspecific competition between two sympatric fish species, *Pennahia argentata* and *Larimichthys polyactis* in Gwangyang Bay, South Korea. Moreover, ontogenetic niche changes were investigated by size classification for each species. The δ^13^C and δ^15^N values in *P. argentata* showed no significant size‐related differences (*p* > 0.1), while *L. polyactis* exhibited significant variation (*p* < 0.001), indicating different ontogenetic niche shifts between two sympatric species. Trophic positions (TP_Glu/Phe_) estimates based on nitrogen isotopic composition of glutamic acid (δ^15^N_Glu_) and that of phenylalanine (δ^15^N_Phe_) were consistent across groups (3.53–3.62), indicating similar diet consumption. However, we found lower δ^15^N_Phe_ values in large *L. polyactis* suggesting assimilated prey from distant marine areas before migrating into the study site. These findings demonstrate the utility of combining bulk stable isotope analysis and amino acid‐specific CSIA to disentangle dietary patterns and habitat use in mobile fish species. This study provides fundamental information for more targeted and ecological management strategies under variable environmental conditions by contrasting ontogenetic niche shifts and habitat‐driven isotopic variation between two sympatric species.

## Graphical abstract

We investigated trophic interactions between two sympatric fish species, *Pennahia argentata* and *Larimichthys polyactis* , using bulk and compound‐specific stable isotope analyses. CSIA of amino acids revealed similar trophic positions but distinct δ^15^N baselines, indicating ontogenetic niche shifts and offshore migration patterns. Our findings demonstrate the utility of CSIA in resolving fine‐scale ecological dynamics in coastal fish communities.

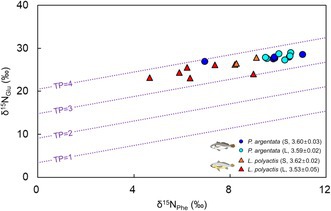

## 1 Introduction

Sympatric species coexist in the same habitat and use similar resources, have ecological similarities, but they often develop differences in habitat use, feeding behavior, and reproductive strategies to reduce interspecific competition (Nie et al. 2019). Thus, understanding their ecological roles and interactions is crucial for developing sustainable fisheries management and conservation strategies. Silver croaker ( *Pennahia argentata* ) and small yellow croaker ( *Larimichthys polyactis* ), both members of the Sciaenidae family, are widely distributed across the Northwestern Pacific, including the East China Sea and the Yellow Sea. These species share both morphological and ecological characteristics, as both species are classified as benthopelagic fish and exhibit similar feeding and spawning habits (Choi et al. 2021; Xue et al. 2005; Chen et al. 2021). For instance, stomach content analysis (SCA) studies have shown that both species feed mainly on macroinvertebrates (e.g., shrimp, crab, and amphipods) and small fishes (Choi et al. 2021; Kang et al. 2022; Kim and Kwak 2022). Both species are also known to inhabit sandy and muddy bottoms from nearshore areas to depths of up to 120 m (Yamaguchi et al. 2006; Lim et al. 2010) and commonly spawn in estuarine and coastal waters shallower than 30 m (Lin et al. 2018; Zhao et al. 2024). However, there are a few notable differences between them: *P. argentata* is a resident species, whereas *L. polyactis* migrates to the coastal waters of the East China Sea and the Yellow Sea for spawning after overwintering in the deep sea (Myoung et al. 2020; Xue et al. 2005). Specifically, *L. polyactis* migrates to the Yellow Sea and the East China Sea following ocean currents for spawning from April to May and then shifts southward for overwintering in early October (Kang et al. 2020; Xiong et al. 2016).

Although *P. argentata* and *L. polyactis* have long been considered commercially valuable species in Korea, China, and Japan, recent declines in their populations may have been driven by human effects including overfishing, climate change, and other environmental stressors (Han et al. 2022; Yamaguchi et al. 2006; Zhang et al. 2022). Specifically, catches of *P. argentata* have decreased markedly from 2574 tons in 1995 to 900 tons in 2010 in Korea (KOSIS 2023). Catches reached a peak of 20,000 tons in the East China Sea during the 1990s but declined drastically to only 22 tons in 2000 (Yamaguchi et al. 2004). Similarly, catches of *L. polyactis* have decreased significantly in Korea from 39,664 tons in 1992 to 7098 tons in the early 2000s. The declines have prompted Northeast Asian countries to implement fisheries management measures, including closed fishing seasons and Total Allowable Catch (TAC) systems, based on comprehensive ecological studies (Ichinokawa et al. 2017; Ding et al. 2023; Lee et al. 2024).

For decades, *P. argentata* has exhibited temporal stability in growth patterns and spawning habits in coastal areas (Yamaguchi et al. 2004, 2006). In contrast, *L. polyactis* has shown significant reductions in average length and age over time due to various stressors, including changes in diet and sea surface temperature (Li et al. 2011; Shan et al. 2017). Furthermore, recent climate change and marine environmental alterations have led to shifts and reductions in coastal spawning areas for *L. polyactis* (Kim et al. 2006; Xiong et al. 2016). For instance, *P. argentata* remains a dominant species in Gwangyang Bay, which serves as a crucial spawning and habitat area for this species, and regarded as a resident species year‐round (Cha and Park 1994; Koh et al. 2014; Lee et al. 2022). Conversely, *L. polyactis* , which traditionally spawn and migrate in the Yellow Sea, has been newly observed in Gwangyang Bay since 2018, suggesting a southward shift in spawning grounds (Liu et al. 2016; Song et al. 2022; Lee et al. 2022). These recent appearances of *L. polyactis* in Gwangyang Bay suggest that the bay may now serve as a newly used spawning and nursery ground for the species. The distributions of *P. argentata* and *L. polyactis* overlap in Gwangyang Bay mainly from late spring to early autumn, coinciding with their peak spawning periods. Moreover, previous studies on gut contents imply that both species continue feeding during this period, suggesting potential for resource competition within Gwangyang Bay (Kang et al. 2022; Kim and Kwak 2022). The climate‐driven habitat shifts observed in *L. polyactis* underscore the need for ecological research into variations in migratory patterns, habitat range, and major spawning grounds. However, the pronounced seasonal migratory behavior of *L. polyactis* presents challenges in clearly elucidating its ecological characteristics.

Stable isotope analysis (SIA) has been extensively used to investigate the ecological characteristics of various species and the relationships among species in an ecosystem. This technique is particularly effective for identifying dietary partitioning and food competition among sympatric species within the same habitat, because carbon isotope (δ^13^C) ratios generally reflect the sources of primary production with relatively small variation, whereas nitrogen isotope (δ^15^N) values exhibit relatively regular fractionation along with a food chain, which can vary dynamically depending on the baseline conditions of the isoscape (Tomaszewicz et al. 2015; Matley et al. 2017; Paez‐Hosas et al. 2020). SIA has also been applied to estimate isotopic niche, providing quantitative information on the ecological niche of various species and possible overlaps, which can be used to infer resource availability of a species and competition among the species in an ecosystem (Faria and Almada 1999; Jackson et al. 2011; Pinnegar and Polunin 1999). It has likewise been employed to track the migration of Chum salmon ( *Oncorhynchus keta* ) over long distances in the Pacific Ocean and to investigate the migration strategies of the demersal fish species brown trout ( *Salmo trutta* ) (Ruokonen et al. 2019; Trueman et al. 2012). At a broader scale, these isotopic approaches are also fundamental for understanding food web processes. Marine food webs are primarily supported by phytoplankton production, with the isotopic composition of carbon and nitrogen in phytoplankton being transferred to higher trophic positions (TP) through the food chain (Trueman et al. 2012). Therefore, the isotopic signatures of primary producers are regarded as baseline indicators (Choi and Shin 2021). Many researchers recognize that the diversity in the stable carbon and nitrogen isotopic baselines presents valuable information regarding the ecology and habitats of aquatic organisms (Barnes et al. 2009; Graham et al. 2010). The isotopic composition of carbon and nitrogen in particulate organic matter (POM), a substitute for phytoplankton in marine ecosystems, varies significantly at the point of assimilation. This variability has been used to trace the movement of marine predators across isotopically distinct regions. For instance, variation in δ^13^C and δ^15^N along bowhead whale ( *Balaena mysticetus* ) baleen plates reflected annual migrations between feeding grounds with different POM baselines (Hobson and Schell 1998), while large‐scale δ^15^N isocapes of particulate organic matter in the Pacific Ocean have been applied to infer regional residency and migration of yellowfin ( *Thunnus albacares* ) and bigeye tuna ( *Thunnus obesus* ) (Graham et al. 2010).

However, performing SIA on bulk tissue may not always be beneficial due to the substantial spatial or temporal variations in isotopic baselines across diverse environments. For instance, δ^15^N values of primary producers within a single marine ecosystem can vary greatly, ranging from 2.4‰ to 10.4‰ in the Mediterranean Sea (Vizzini et al. 2002). These large range of δ^15^N values in primary producer is caused by the isotopic ratio and the quantity of utilized nitrogenous nutrients. Moreover, the rapid tissue turnover rates in phytoplankton are sensitive to variations in environmental nitrogen sources, while the relatively longer tissue turnover rates in higher trophic organisms reflect time‐integrated nitrogen sources. These differences in temporal integration among organisms reflect time‐integrated nitrogen sources. These differences in temporal integration among organisms can sometimes lead to the misestimation of TP based on the nitrogen isotope analysis in bulk tissue (Choi and Shin 2021). In particular, for fish with long life histories, where tissue turnover rates can vary from months to several years, the use of bulk SIA in ecological studies requires an appropriate baseline (Hesslein et al. 1993; Post 2002). Furthermore, the trophic discrimination factor, typically assumed to be 3.4‰ for nitrogen, can also vary considerably among consumer species and even among tissues within a single individual, often influenced by environmental conditions such as temperature (Choi et al. 2017).

In contrast, compound‐specific stable isotope analysis (CSIA) of individual amino acids has gained attention from ecologists because it can provide accurate estimates of both δ^15^N_baseline_ and TP (Choi et al. 2017; McMahon et al. 2013). Trophic amino acids (e.g., glutamic acid, aspartic acid, alanine, leucine, isoleucine, proline, and valine) undergo isotopic fractionation during transamination and deamination, resulting in a significant isotopic difference of over 5‰ between consumer and diet (McMahon et al. 2015). Among these amino acids, glutamic acid (Glu) is the most enriched in ^15^N, playing a crucial role as the primary nitrogen shuttle for protein synthesis within the food web and exhibiting the largest trophic fractionation with TP shifts (McCarthy et al. 2013; White 2007). In contrast, the δ^15^N of source amino acids, such as methionine, tyrosine, and phenylalanine, shows little difference between diets and consumers. Among them, phenylalanine (Phe) is known to have the most invariant δ^15^N during trophic transfer (Chikaraishi et al. 2007, 2009). Based on these isotopic characteristics, diverse studies established a TP calculation method (TP_Glu/Phe_), which has been employed across various taxonomic groups (Germain et al. 2013; Chikaraishi et al. 2014; Bradley et al. 2015; Nielsen et al. 2015).

Observations of *L. polyactis* recently entering Gwangyang Bay, a habitat traditionally dominated by *P. argentata* , suggest a climate‐driven southward shift in its distribution. This change necessitates a clear understanding of the size‐dependent ecological niche interactions between these two sympatric species. Despite this need, information directly comparing the ecological characteristics of these coexisting species in the same habitat is scarce. Therefore, this study aims to elucidate the size‐based niche relationship between *L. polyactis* and *P. argentata* in Gwangyang Bay. We applied bulk tissue stable isotope analysis (SIA) to estimate the isotopic niche and compound‐specific isotope analysis (CSIA) of amino acids to estimate trophic position (TP). This integrated approach aims to enhance the understanding of ecological interactions between these sympatric species and highlights the utility of combined SIA and CSIA for obtaining precise ecological information.

## 2 Materials and Methods

### 2.1 Study Sites and Sampling

**FIGURE 1:**
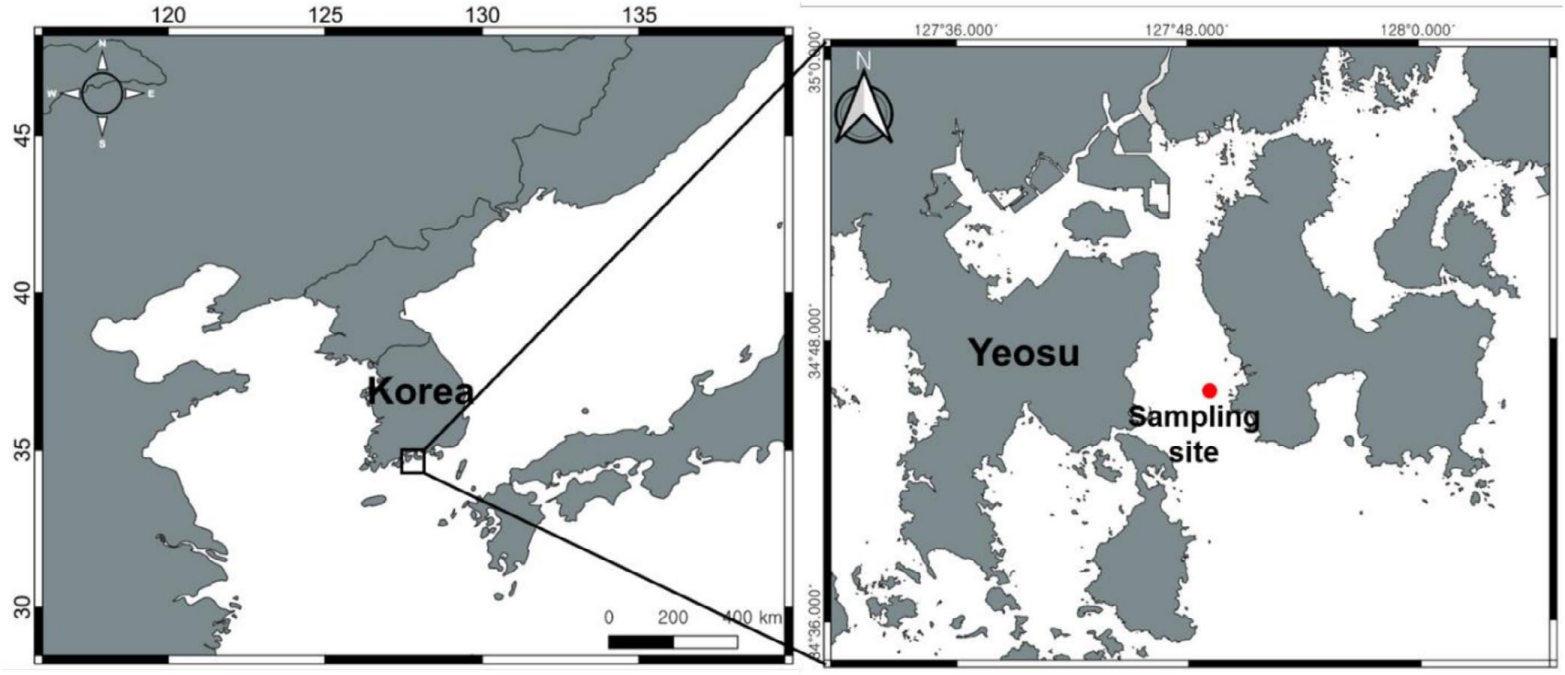
Sampling location of *Pennahia argentata* and *Larimichthys polyactis* from Gwangyang bay in South Korea (127°49.5 E, 34°45.7 N).

Fish sampling was conducted in September 2020 at the entrance of Gwangyang Bay, which is located in the central part of the southern coast of the Korean Peninsula (Figure 1). This semi‐enclosed bay is connected to the Seomjin Estuary, which contributes to a substantial inflow of nutrients and supports a high level of species diversity. Additionally, it is an ecologically significant area for numerous fish species, serving as spawning grounds, nursery habitats, and feeding grounds.

All fish utilized in this study were acquired from local commercial fishers as part of routine fishing operations and were already deceased prior to research activities. Consequently, the authors did not handle any live animals nor perform any procedures that could cause pain or distress. Based on institutional guidelines, this type of activity was exempt from Institutional Animal Care and Use Committee (IACUC) approval.

A shrimp beam trawl (length: 8 m, width: 8 m, mesh size of wing and body: 3 cm, mesh line: 1 cm) was towed for 10 min at 1–2 knots at the sampling site. Collected fish samples were measured for total length (TL), and then immediately preserved at −80°C using dry ice until they were transported to the laboratory. Based on TL, 142 individuals of *P. argentata* were categorized into a small group (0–1 years old, TL < 187 mm, *n* = 93) and a large group (≥ 2 years old, TL > 188 mm, *n* = 49) (Kwon et al. 1999). Similarly, 59 individuals of *L. polyactis* were categorized into a small group (0–1 years old, TL < 160 mm, *n* = 15) and a large group (≥ 2 years old, TL > 160 mm, *n* = 44) (Lee et al. 2000). *P. argentata* typically reaches sexual maturity after one year, with a total length of approximately 160–190 mm. *L. polyactis* begins active seasonal migration for spawning and overwintering from two years of age. Therefore, we categorized individuals into two groups: presumed 0–1 year‐olds (small group) and 2+ year‐olds (large group).

### 2.2 Stable Isotope Analysis

Dorsal muscle tissue was dissected from each fish specimen. The extracted dorsal muscles were freeze‐dried and homogenized. Approximately 1 mg of each sample was sealed in a tin capsule for carbon and nitrogen SIA. The SIA was performed using an isotope ratio mass spectrometer (Isoprime VisION IRMS, Elementar, Manchester, UK) linked with an elemental analyzer (vario ISOTOPE cube, Elementar, Langenselbold, Germany). Typically, removing lipids is necessary for carbon SIA. However, this process was omitted in this study because the dorsal muscle samples had low lipid content, as a mean C:N ratio of 3.4, which is below the commonly applied threshold of 3.5 for lipid removal requirement (Post et al. 2007; Logan et al. 2008; von Biela et al. 2016). The measured stable isotope ratios were expressed as ‘δ’ values, calculated using the following formula (Equation 1):

(1) $$\delta^{13}C\;\text{or}\;\delta^{15}N=\left[\left(R_{\text{sample}}/R_{\text{standard}}\right)-1\right]\times 1000,$$

where *R* = ^13^C/^12^C for carbon isotopes and ^15^N/^14^N for nitrogen isotopes.

Vienna Pee Dee Belemnite (VPDB) and atmospheric nitrogen gas were used as standard materials for δ^13^C and δ^15^N stable isotope calculations, respectively. The standards CH‐3 (δ^13^C, −24.72‰ ± 0.1‰) and N‐1 (δ^15^N, 0.4‰ ± 0.1‰), provided by the International Atomic Energy Agency (IAEA), were analyzed after every five samples to ensure analytical accuracy. Analytical error was confirmed to be within ±0.1‰ for δ^13^C and ±0.2‰ for δ^15^N.

The derivatization process for CSIA of the nitrogen in amino acids followed the procedure described by Chikaraishi et al. (2007). Briefly, approximately 1 mg of homogenized dorsal muscle was hydrolyzed with 12 M HCl for 12 h at 110°C, and hydrophobic materials were removed using a mixture of *n*‐hexane and dichloromethane (3:2, v/v). Isopropyl esterification and pivaloylation were then performed for 2 h at 110°C using mixtures of thionyl chloride and 2‐propanol (1:4, v/v) and pivaloyl chloride and dichloromethane (1:4, v/v), respectively. The δ^15^N values of individual amino acids were determined using an isotope ratio mass spectrometer (Isoprime 100, Isoprime, U.K.) coupled with a gas chromatograph (Agilent Technologies 6890 N, Agilent, U.S.).

The TPs of *P. argentata* and *L. polyactis* were calculated based on the nitrogen isotopic composition of glutamic acid (δ^15^N_Glu_) and phenylalanine (δ^15^N_Phe_) using the following formula (Equation 2):

(2) $${\mathrm{TP}}_{\mathrm{Glu}/\mathrm{Phe}}=\left[\left(\delta^{15}N_{\mathrm{Glu}}-\delta^{15}N_{\mathrm{Phe}}-\beta \right)/\mathrm{TDF}\right]+1,$$

where *β* was set to 3.4‰ to account for the consistent offset between δ^15^N_Glu_ and δ^15^N_Phe_ in primary producers, and TDF was set to 5.7‰ to account for the trophic discrimination factors of fish samples (Bradley et al. 2015).

### 2.3 Statistical Analysis

R statistical software (version 2021.09.1, Posit PBC, Redmond, WA, USA) was used for statistical analyses in this study. A one‐way analysis of variance was conducted to test for significant differences in δ^13^C and δ^15^N among the different size classes of *P. argentata* and *L. polyactis* . Significant differences were determined using Tukey's test, with a *p*‐value threshold of less than 0.05.

The standard ellipse area corrected (SEAc) for small sample sizes represents the core isotopic niche space of *P. argentata* and *L. polyactis* . SEAc was calculated using the Stable Isotope Bayesian Ellipses in R (SIBER) package (Jackson et al. 2011, 2012). To test for differences in isotopic niche width among groups, we generated posterior distributions of SEAc using 10,000 bootstrap resamples and calculated the posterior probability (*Pr*) that one group had a larger SEAc than another. A probability threshold of *Pr* > 0.95 was considered as evidence for a significant difference. Subsequently, the extent of overlap in the trophic space of the standard ellipse areas for each group was assessed. A higher proportion of overlapping areas indicates a greater similarity in diet between the two groups.

## 3 Results

### 3.1 The Distribution of Fish in Gwangyang Bay and the Total Length Frequency of *Pennahia argentata* and *Larimichthys polyactis*

**FIGURE 2:**
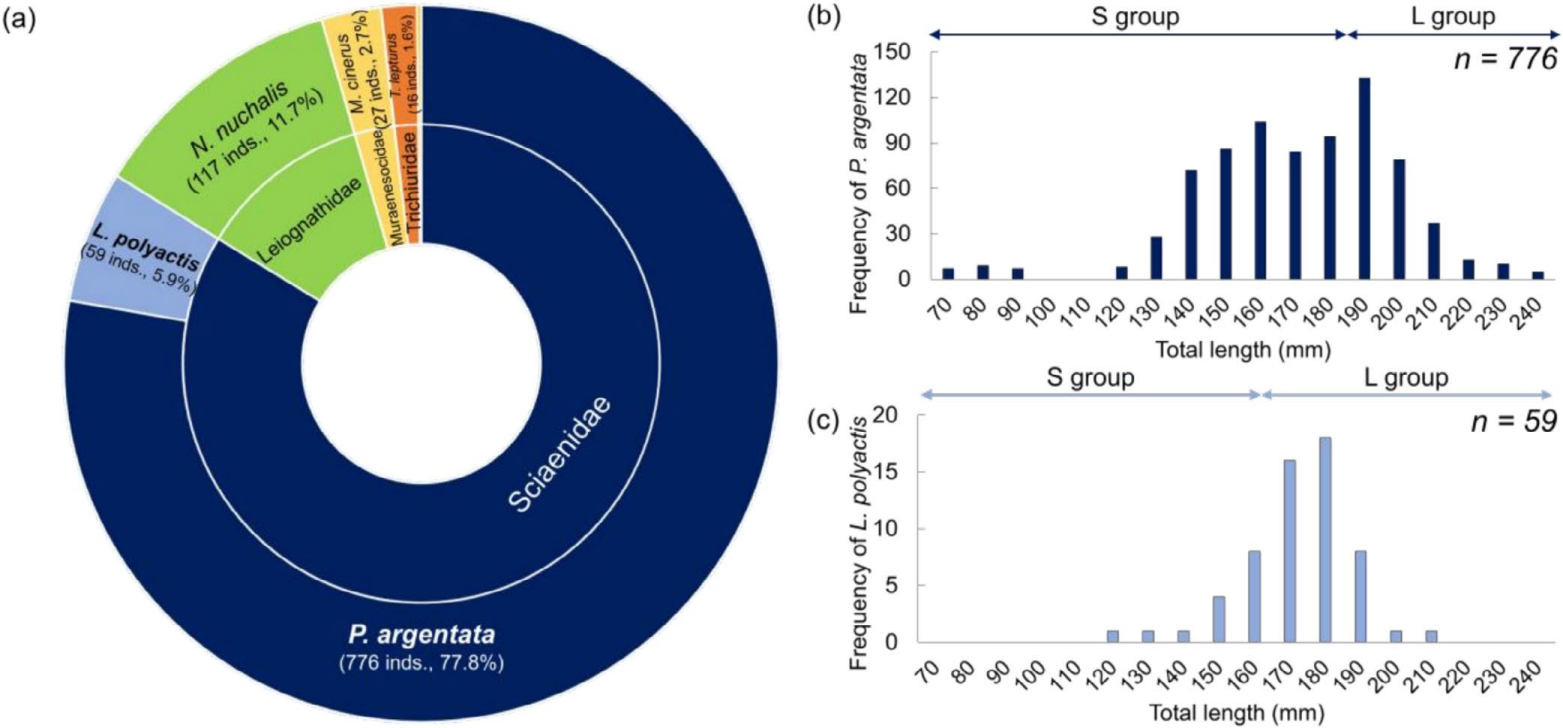
Fishery data collected from Gwangyang Bay, South Korea, in September 2020. (a) Sunburst chart of fish collected from Gwangyang Bay, showing the proportion of different fish species. The inner circle represents the family, and the outer circle represents the species. (b) Distribution of total length for *Pennahia argentata* (*n* = 776), indicating the frequency of individuals within specific length intervals. (c) Distribution of total length for *Larimichthys polyactis* (*n* = 59), indicating the frequency of individuals within specific length intervals.

A total of 997 individuals representing six species were collected, with *P. argentata* being the dominant species, accounting for 77.8% of the relative abundance (Figure 2a). Spotnape ponyfish (*Nuchequula nuchalis*) was the subdominant species, with 117 individuals (11.7%) collected, while *L. polyactis* , also from the Sciaenidae family, accounted for 59 individuals (5.9%) of the collected specimens.

The TL of *P. argentata* ranged from 62 to 240 mm, while that of *L. polyactis* ranged from 120 to 210 mm. Based on age, 151 individuals of *P. argentata* were classified as under one year old, 457 as two years old, and 168 as three years old or older. In terms of TL frequency, the highest number of individuals was observed in the 180–190 mm TL range (133 individuals), while the lowest frequency was recorded in the 230–240 mm TL range (5 individuals) (Figure 2b). For *L. polyactis* , 3 individuals were classified as under one year old, 14 as two years old, and 42 as three years old or older. Furthermore, the highest number of individuals was observed in the 170–180 mm TL range (18 individuals), followed by 16 individuals in the 160–170 mm TL range. Other size groups had under ten individuals.

### 3.2 Carbon and Nitrogen Isotope Ratios in Bulk Tissues Extracted From Dorsal Muscles

**FIGURE 3:**
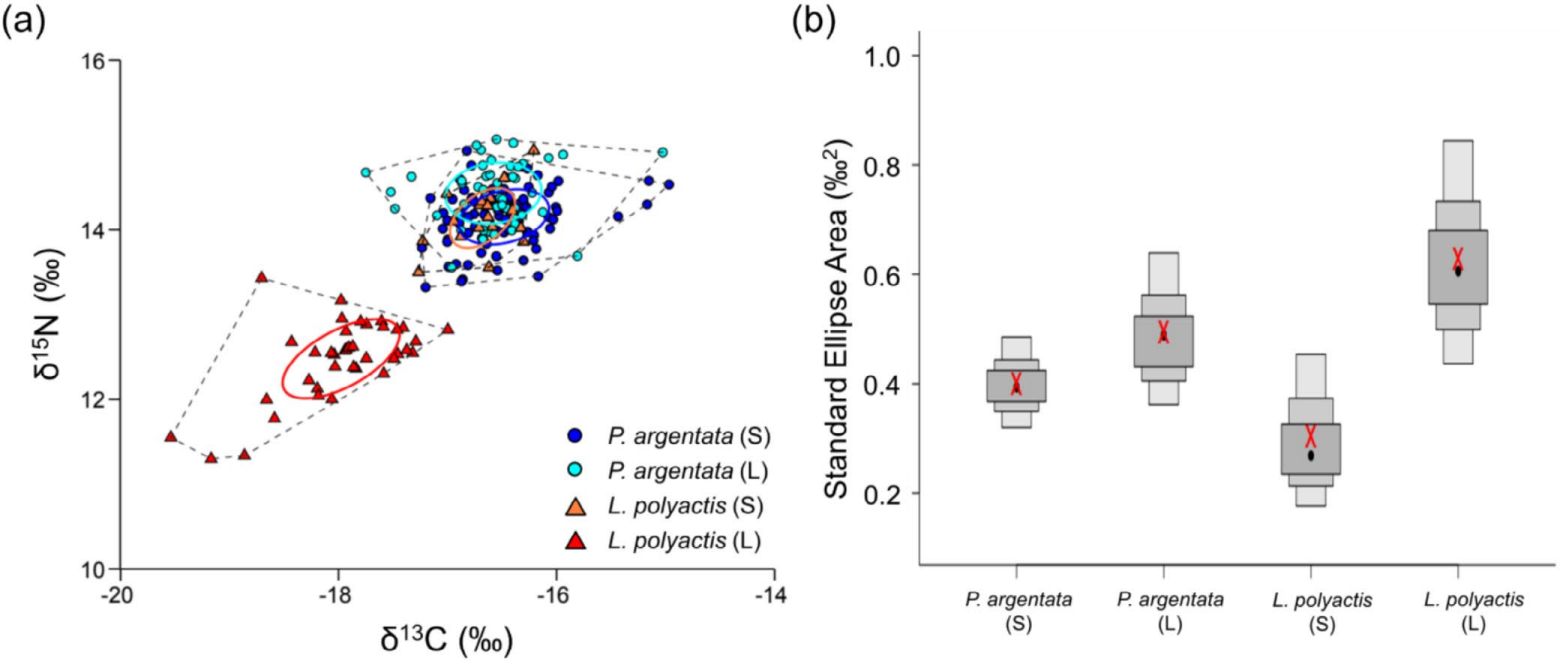
Isotopic niches of *P. argentata* and *L. polyactis* . (a) Variations in δ^13^C and δ^15^N values for *P. argentata* and *L. polyactis* from Gwangyang Bay. Solid lines and dotted lines represent the 95% CI bivariate ellipses and convex hulls estimated using Stable Isotope Bayesian Ellipses in R (SIBER) analysis, respectively. (b) Density plots showing the credibility intervals (95%, 75%, and 50%) of the standard ellipse areas for *P. argentata* and *L. polyactis* in Gwangyang Bay, estimated using SIBER analysis. Black dots represent the mean standard ellipse area, while red ‘X’ represents the mean standard ellipse areas corrected (SEAc) for small sample sizes.

The stable isotope ratios for *P. argentata* ranged from −17.8‰ to −15.0‰ for δ^13^C and from 13.3‰ to 15.1‰ for δ^15^N (Figure 3a). *Larimichthys polyactis* exhibited δ^13^C values ranging from −19.5‰ to −16.1‰ and δ^15^N values ranging from 11.3‰ to 14.9‰, showing partially overlapping but overall more depleted isotope values compared to *P. argentata* . The mean δ^13^C and δ^15^N values for *P. argentata* were −16.52‰ ± 0.07‰ and 14.24‰ ± 0.06‰ (95% CI), respectively, while *L. polyactis* showed lower mean values of −17.53‰ ± 0.20‰ for δ^13^C and 13.01‰ ± 0.22‰ for δ^15^N (95% CI). These confidence intervals indicate some overlap between the two species but also reveal that *L. polyactis* generally occupies a more δ^13^C‐ and δ^15^N‐depleted isotopic space, and the differences were statistically significant for both isotope values (Welch's *t*‐test, δ^13^C: *p* < 0.001; δ^15^N: *p* < 0.001).

Regarding size‐based δ^13^C and δ^15^N ratios, *L. polyactis* (L) formed a distinct niche, while *P. argentata* (S and L groups) and *L. polyactis* (S) exhibited similar values (Figure 3a). One‐way ANOVA results showed a significant difference in δ^13^C and δ^15^N stable isotope ratios for *L. polyactis* (L) (*p* < 0.01) (Table 1). However, no significant differences in carbon and nitrogen isotope ratios were observed among the other three groups (*p* > 0.1).

**TABLE 1:** Average carbon and nitrogen isotope ratios for *Pennahia argentata* and *Larimichthys polyactis* by size. “S” denotes small individuals, typically aged 1–2 years, while “L” represents large individuals, typically aged 3 years or older. Significant differences between each group are presented at *p < 0.05.

Species and size	No. of samples	δ^13^C	δ^15^N
*P. argentata* (S)	93	−16.5 ± 0.0	14.1 ± 0.0
*P. argentata* (L)	49	−16.6 ± 0.1	14.4 ± 0.1
*L. polyactis* (S)	15	−16.8 ± 0.1	14.0 ± 0.2
*L. polyactis* (L)	44	−17.8 ± 0.1*	12.7 ± 0.1*

Isotopic niche areas for the four groups of *P. argentata* and *L. polyactis* , as delineated by SEAc, are presented in Figure 3b. The *P. argentata* (S and L) and *L. polyactis* (S) groups were positioned relatively higher in δ^13^C values within the δ‐space than the *L. polyactis* (L) group, which exhibited greater depletion in both δ^13^C and δ^15^N values. The total area (TA) was in the following order: *P. argentata* (L, 2.68‰^2^) > *L. polyactis* (L, 2.65‰^2^) > *P. argentata* (S, 2.28‰^2^) > *L. polyactis* (S, 0.92‰^2^). The SEAc of *P. argentata* increased from 0.40‰^2^ in the S group to 0.49‰^2^ in the L group, but this difference was not statistically significant (*Pr* = 0.76). In contrast, *L. polyactis* exhibited a significant niche expansion from 0.30‰^2^ in the S group to 0.63‰^2^ in the L group (*Pr* = 0.98), indicating a widening niche with growth. The δ‐space of *L. polyactis* (L) was distinct and did not overlap with that of the other three groups. In contrast, the areas of overlap were 0.29‰^2^ between *P. argentata* (S) and *P. argentata* (L), 0.23‰^2^ between *P. argentata* (S) and *L. polyactis* (S), and 0.18‰^2^ between *P. argentata* (L) and *L. polyactis* (S).

### 3.3 Trophic Positions by Group Based on Compound‐Specific Amino Acid Analysis

**FIGURE 4:**
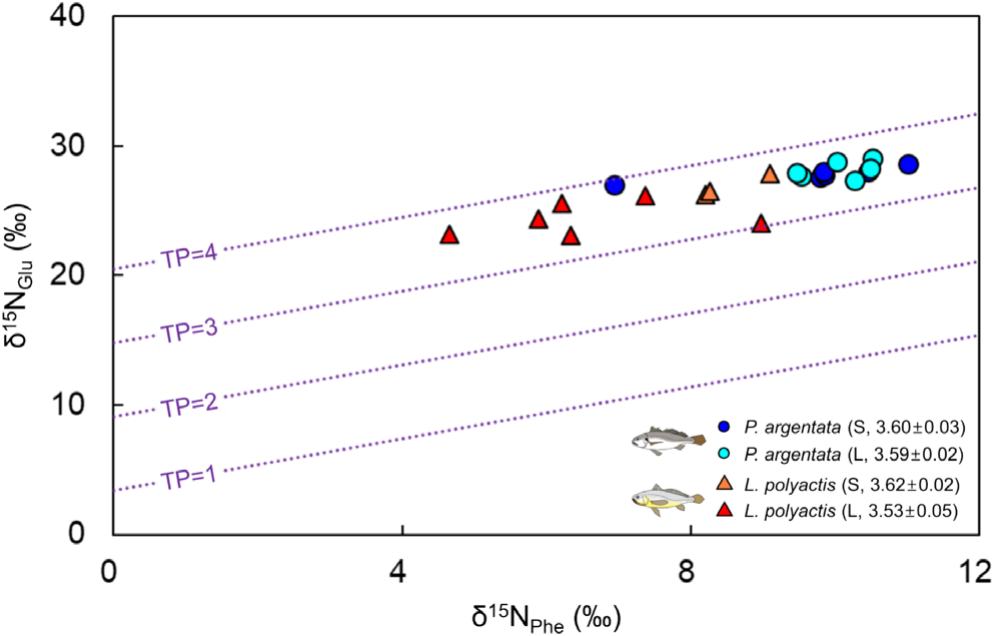
Amino acid isotope compositions of the δ^15^N values of glutamic acid (Glu) and phenylalanine (Phe). Lines represent integer trophic positions (TPs, i.e., 1, 2, 3, and 4), estimated using Equation (2). Numbers in brackets represent the mean ± SD of TP for the four groups. Numbers in brackets represent the mean ± standard error (SE) of TP for the four groups. The SE appears as 0.0 due to rounding to one decimal place.

The δ^15^N_Glu_ and δ^15^N_Phe_ values were plotted along a trophic gradient (trophocline) described by Equation (2) (Figure 4). For *P. argentata* , δ^15^N_Glu_ ranged from 27.0‰ to 29.1‰ (average 28.0‰ ± 0.2‰), and δ^15^N_Phe_ ranged from 6.9‰ to 11.0‰ (average 9.8‰ ± 0.3‰). In contrast, *L. polyactis* exhibited δ^15^N_Glu_ values ranging from 23.1‰ to 27.8‰ (average 25.2‰ ± 0.5‰) and δ^15^N_Phe_ values ranging from 4.6‰ to 9.1‰ (average 7.2‰ ± 0.5‰). The highest mean values of δ^15^N_Glu_ and δ^15^N_Phe_ were observed in *P. argentata* (L) followed by *P. argentata* (S) and *L. polyactis* (S). Conversely, *L. polyactis* (L) exhibited the lowest δ^15^N_Glu_ and δ^15^N_Phe_ values, with significant differences observed across all groups (*p* < 0.01). The estimated TP based on the δ^15^N_Glu_ and δ^15^N_Phe_ values (TP_Glu/Phe_) was similar among the four groups, with mean values of 3.60 and 3.59 for *P. argentata* (S and L groups, respectively) and 3.62 and 3.53 for *L. polyactis* (S and L groups, respectively), and no significant differences were detected among groups (*p* > 0.5).

## 4 Discussion

The distribution and TL frequency analysis of sympatric fish in Gwangyang Bay offer valuable insights into the size structure and stage‐specific habitat use of *P. argentata* and *L. polyactis* , thereby contributing to a better understanding of their ecological population dynamics and habitat preferences. The dominance of *P. argentata* observed in this study, together with previous reports of its long‐term prevalence in Gwangyang Bay (Han et al. 2019; Lee et al. 2022; Choi et al. 2021), highlights its sustained ecological significance in the region. The TL frequency data reveal a well‐established age structure for *P. argentata* , which is generally indicative of a stable and healthy reproducing population, as commonly inferred in fish population studies (King 2013; Shin et al. 2005). The presence of a substantial number of individuals across various age groups, including a significant number of three‐year‐old and older individuals, suggests successful recruitment and growth within the bay (Kim and Kwak 2022). In contrast, the length frequency distribution of *L. polyactis* shows a narrower range with fewer individuals, particularly in the smaller size groups. This pattern may suggest a less stable population structure compared to *P. argentata* , but it should be interpreted with caution given the relatively small sample size and the single sampling event. This pattern may also be linked to recent southward shifts in spawning grounds under warming conditions, which have been reported for *L. polyactis* (Han et al. 2021). Overall, the dominance and consistent presence of *P. argentata* in contrast with the more limited occurrence of *L. polyactis* observed in this study indicate differences in their ecological roles and habitat use. Therefore, future management strategies should incorporate spatiotemporal patterns of age structure, and annual monitoring surveys will be essential for accurately tracking shifts in population composition of the two species within Gwangyang Bay.

SIA has been widely employed to provide a temporally integrated perspective on the dietary relationships of organisms (Vander Zanden et al. 1999). The minor difference in δ^13^C values between dietary resources and consumers provides information on diet and geographic habitat, whereas the stepwise enrichment of δ^15^N along the food chain reflects trophic position (DeNiro and Epstein 1978; Minagawa and Wada 1984). Therefore, the breadth of δ^13^C and δ^15^N values is commonly used as an isotopic niche space, and comparisons within species are often used to infer ontogenetic diet shift (Davis et al. 2012). The more depleted δ^13^C values of the L group of *L. polyactis* than those of the S group shown in the present study can be derived from reflecting offshore‐originating isotopic baselines assimilated prior to migration into Gwangyang Bay. This interpretation is consistent with the migratory behavior of *L. polyactis* , suggesting that individuals in the L group foraged in offshore habitats before entering the bay while the S group likely assimilated carbon from local coastal food webs (Wang et al. 2016; Song et al. 2023). In the case of *P. argentata* , based on their resident life cycle that spawns and grows within a bay, individuals should exhibit carbon isotopic signatures that reflect the local coastal baseline. Theerefore, similar δ^13^C values of S group of *L. polyactis* with *P. argentata* shown the present study supports the interpretation that the relatively enriched δ^13^C values of *L. polyactis* (S) reflect residency within the estuarine and coastal environment of Gwangyang Bay.

Notably, the isotopic niche width of the L groups of *L. polyactis* was substantially larger than that of their respective S groups (Figure 3b), indicating niche expansion in habitat use and dietary sources with growth. This niche expansion is ecologically meaningful, as it likely reflects exposure to multiple environments with isotopically distinct baselines. The expansion of isotopic niche width of this species may be attributed to its life history strategy of residing in estuarine habitats during early stages and expanding habitat use through offshore migration upon reaching a certain age (Song et al. 2023). Interestingly, despite isotopic niche width expansion, the TP_Glu/Phe_ remained relatively consistent across size groups, indicating that the observed niche expansion is not necessarily driven by increased consumption of higher trophic level prey, but rather by greater dietary or habitat diversity. This decoupling of trophic position and niche width suggests that both species may exhibit ecological plasticity in response to environmental variability, such as seasonal or spatial changes in prey availability. In contrast, the diet of *P. argentata* was consistently dominated by decapods (e.g., *Crangon affinis* ) across all size classes (Koh et al. 2014). Although larger individuals tended to consume a wider range of prey including small fishes such as anchovy ( *Engraulis japonicus* ), no significant ontogenetic diet shift was reported. Moreover, an integrated SIA and SCA study conducted in Gwangyang Bay also indicated an increase in isotopic niche width with body size. This increase was consistent with a gradual broadening of prey composition, from mainly Crustacea in early life stages to a more diverse prey diversity assemblage including Decapoda and Isopoda in later stages, a pattern also observed in the present study (Choi et al. 2021).

SCA studies have demonstrated that *L. polyactis* undergoes an ontogenetic diet shift, showing dietary patterns similar to *P. argentata* during early life stages and increasing its reliance on anchovy as it grows (Kang et al. 2022; Xue et al. 2005). However, in the present study, the L group of *L. polyactis* exhibited relatively lower δ^15^N values than the S group, which contradicted our expectations. Typically, increased consumption of higher TP diets (e.g., fish) is accompanied by δ^15^N trophic enrichment in the consumer (Schmidt et al. 2007). Several factors may explain our unexpected pattern, including the size structure of the sampled individuals and differences in isotopic baselines. Kang et al. (2022) suggested that ontogenetic diet shifts in *L. polyactis* occur up to 200 mm in TL, which is larger than the size range of the L group individuals analyzed in this present study. Therefore, due to the insufficient number of individuals larger than 200 mm TL, we may not have captured the full extent of diet shift. Second, the unexpected δ^15^N patterns may be driven by differences in nitrogen isotopic baselines between groups rather than dietary differences.

CSIA of amino acids enhances the accuracy and precision of TP estimates compared to the bulk tissue approach (Blanke et al. 2017). This method is particularly useful for fish species that roam across vast areas and consume a wide variety of prey, as it allows for the estimation of TP of organisms within an ecosystem without requiring the isotopic information of prey organisms (Choi et al. 2020; Pollierer et al. 2019). In this study, the mean TP_Glu/Phe_ values of all groups ranged from 3.53 to 3.62, indicating feeding on a mixed diet of primary consumers (TP = 2), such as zooplankton, and secondary consumers (TP = 3), such as benthic organisms and small fish. The trophocline, depicted using δ^15^N values of trophic and source amino acids on the x‐ and y‐axes, respectively, can be used not only for indicating TP but also for understanding isotopic baselines and food web structures (Xing et al. 2020). The δ^15^N_Phe_ values are particularly useful for elucidating isotopic baselines because they exhibit minimal trophic discrimination between diet and consumer (Chikaraishi et al. 2014). Notably, δ^15^N of a consumer reflects the nitrogen isotopic baseline of primary producers in a given habitat, and this is because nitrogen sources differ substantially across regions (Minagawa and Wda 1986; Altabet and Francois 1994). For instance, estuaries and coastal areas influenced by riverine input of inorganic nitrogen typically exhibit higher δ^15^N baselines, which tend to decrease as one moves toward offshore areas (Krause et al. 2023). Accordingly, δ^15^N_Phe_ values can be variable among fish species depending on their habitat.

This proxy has not only been used to trace migratory pathways in marine fishes (Matsubayashi et al. 2020), but also serves as a tool to distinguish habitat use between coastal and offshore species. For example, fish inhabiting offshore environments generally exhibit lower δ^15^N_Phe_ values due to depleted nitrogen isotopic baselines, while coastal species tend to have higher values. Migratory species that traverse both regions typically show a wider range of δ^15^N_Phe_ values (Xing et al. 2020). Thus, based on the migrating lifestyle from offshore to coastal habitats, the wide range of δ^15^N_Phe_ values observed in *L. polyactis* can be derived from spatial variation. Despite the wide range of δ^15^N_Phe_ in *L. polyactis* , TP values remained consistent, indicating that the larger isotopic niche width in the L group reflects greater variability in geological habitat use and prey diversity rather than a shift to higher trophic‐level feeding. This finding underscores the importance of using CSIA in conjunction with bulk SIA, as it enables researchers to resolve the confounding effects of environmental isotopic variation and accurately interpret ecological patterns. This pattern was not observed in *P. argentata* , a resident species, which showed relatively uniform δ^15^N_Phe_ values.

Previous isotopic studies on *L. polyactis* across five regions of China coasts revealed clear spatial separation among populations based on δ^18^O and δ^13^C values, suggesting ecological differentiation associated with mobility (Wang et al. 2016). In addition, *L. polyactis* undergoes annual migration beginning at 2–3 years of age, after spending its early life stages in coastal nursery habitats, thus experiencing environmentally heterogeneous habitats throughout its life history (Song et al. 2023). A bulk SIA study conducted in Chinese coastal waters found lower δ^13^C values in mature individuals (126–185 mm body length), which was interpreted as a shift toward more pelagic feeding sources (Weiwei et al. 2011). However, this pattern was primarily observed in comparisons between juveniles (60–80 mm) and larger individuals, whereas δ^15^N values showed no significant differences among mature fish. Those previous studies support our interpretation that the relatively low δ^13^C values, distinct niche plot, and large range of δ^15^N_Phe_ values in the L group of *L. polyactis* may reflect isotopic baselines derived from offshore habitats associated with the migratory behavior of individuals older than two years. Furthermore, the consistent trophic positions (TP 3.53–3.62) observed across all groups indicate an absence of size‐related dietary shifts within *L. polyactis* and imply potential dietary overlap between *P. argentata* and *L. polyactis* in Gwangyang Bay. Consequently, our results support the view that the migratory and spawning ground patterns of *L. polyactis* have been undergoing shifts, offering new evidence of ecological change in this species.

This study demonstrates that combining bulk SIA and compound‐specific CSIA provides a more nuanced understanding of trophic relationships and habitat use than either method alone. By integrating both approaches, we were able to disentangle the effects of dietary variation and isotopic baseline shifts, offering clearer ecological interpretations for species with complex life histories. Such combined isotopic analyses are particularly valuable in coastal ecosystems where baseline variability and species mobility complicate trophic assessments. This integrative framework strengthens the ecological basis for fisheries management and supports the development of more precise, adaptive conservation strategies.

## Author Contributions

**Tae‐Sik Yu:** data curation (equal), formal analysis (equal), visualization (equal), writing – original draft (equal). **Bohyung Choi:** conceptualization (equal), methodology (equal), writing – review and editing (equal). **Yeonjung Lee:** formal analysis (equal). **Ihn‐Sil Kwak:** conceptualization (equal), funding acquisition (equal), methodology (equal), project administration (equal), supervision (equal), validation (equal), writing – review and editing (equal).

## Conflicts of Interest

The authors declare no conflicts of interest.

## Supporting information


**Data S1:** Supporting information.

## Data Availability

All data are available as Supporting Information in three Excel sheets (S1a to S1c). S1a: Length, weight, and bulk carbon and nitrogen stable isotope data for *Pennahia argentata* . S1b: Length, weight, and bulk carbon and nitrogen stable isotope data for *Larimichthys polyactis* . S1c: Compound‐specific stable isotope analysis (CSIA) and trophic position (TP_Glu/Phe_) estimates for *Pennahia argentata* and *Larimichthys polyactis* , categorized by groups.
